# High-level production of poly-γ-glutamic acid from untreated molasses by *Bacillus siamensis* IR10

**DOI:** 10.1186/s12934-020-01361-w

**Published:** 2020-05-12

**Authors:** Dexin Wang, Hyangmi Kim, Sungbeom Lee, Dae-Hyuk Kim, Min-Ho Joe

**Affiliations:** 1 Radiation Utilization and Facilities Management Division, Korea Atomic Energy Research Institute, 29 Geumgu-gil, Jeongeup, 56212 Republic of Korea; 2 Department of Bioactive Material Sciences, Institute for Molecular Biology and Genetics,Center for Fungal Pathogenesis, Jeonbuk National University, Jeonju, 54896 Republic of Korea; 3 Bacteria Research Team, Nakdonggang National Institute of Biological Resources (NNIBR), Sangju, 37242 Republic of Korea; 4 Radiation Research Division, Korea Atomic Energy Research Institute, 29 Geumgu-gil, Jeongeup, 56212 Republic of Korea; 5 Department of Radiation Science and Technology, University of Science and Technology, Daejeon, 34113 Republic of Korea

**Keywords:** *Bacillus siamensis* IR10, Gamma irradiation, Poly-γ-glutamic acid, Untreated molasses, Fermentation

## Abstract

**Background:**

Poly-γ-glutamic acid (γ-PGA) is a promising biopolymer and has been applied in many fields. *Bacillus siamensis* SB1001 was a newly isolated poly-γ-glutamic acid producer with sucrose as its optimal carbon source. To improve the utilization of carbon source, and then molasses can be effectively used for γ-PGA production, ^60^cobalt gamma rays was used to mutate the genes of *B. siamensis* SB1001.

**Results:**

*Bacillus siamensis* IR10 was screened for the production of γ-PGA from untreated molasses. In batch fermentation, 17.86 ± 0.97 g/L γ-PGA was obtained after 15 h, which is 52.51% higher than that of its parent strain. Fed-batch fermentation was performed to further improve the yield of γ-PGA with untreated molasses, yielding 41.40 ± 2.01 g/L of γ-PGA with a productivity of 1.73 ± 0.08 g/L/h. An average γ-PGA productivity of 1.85 g/L/h was achieved in the repeated fed-batch fermentation. This is the first report of such a high γ-PGA productivity. The analysis of the enzyme activities showed that they were affected by the carbon sources, enhanced ICDH and GDH, and decreased ODHC, which are important for γ-PGA production.

**Conclusion:**

These results suggest that untreated molasses can be used for economical and industrial-scale production of γ-PGA by *B. siamensis* IR10.
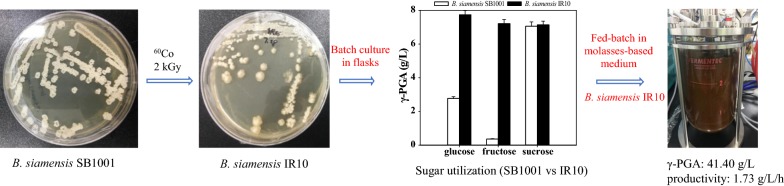

## Background

Poly-γ-glutamic acid (γ-PGA) is a high molecular biopolymer composed of glutamic acid repeating units with gamma-amide linkages [1]. The characteristics of γ-PGA such as excellent water solubility, biodegradability, super absorption, and nontoxicity contribute to a wide range of applications in many fields [2–4]. The high water absorption corresponds with a high viscosity and leads to applications such as a thickener in the food industry [5] and a moisturizer in cosmetics [6]. Moreover, γ-PGA has great applications in other fields, including in drug delivery in medicine [7], biopolymer flocculant adsorption of toxic metal ions used in the sewage treatment industry [2, 8], regulation of the soil micro-environment in agriculture, improvement of nitrogen use efficiency, and promotion of plant growth [9].

Most *Bacillus* strains, including *B. subtilis*, *B. licheniformis*, *B. amyloliquefaciens*, *B. methylotrophicus* and *B. megaterium*, have been reported to produce γ-PGA from glucose, glycerol, sucrose, and fructose based media [10–14]. Until now, intensive studies regarding the optimization of the medium composition and fermentation conditions [10, 15], metabolic regulation [16, 17], and genetic engineering methods [18, 19] have been done to improve the yield of γ-PGA. Due to the increasing applications of γ-PGA, low-cost substrates and high productivity are essential for the commercial production of γ-PGA in addition to increasing the γ-PGA yield. Inexpensive substrates, such as molasses, a by-product of refining sugarcane, containing high sugar concentrations and several dietary minerals necessary for cell growth and fermentation, already are used for the production of polysaccharides including γ-PGA [20]. Additionally, rice straw and corncob fibers hydrolysates also have been investigated for the production of γ-PGA [21, 22]. Although these low-cost substrates produce a certain amount of γ-PGA, the productivity is not satisfactory, which is lower than 1.0 g/L/h. Thus, when trying to achieve low cost and high productivity, the choice of an efficient γ-PGA producing strain is essential.

In this study, a *Bacillus siamensis* SB1001 mutant strain was isolated and named *B. siamensis* IR10, which can use a variety of sugars including molasses to produce a high amount of γ-PGA. To explore the mechanism of the mutation, the key enzyme activities at the 2-oxoglutarate branch were determined in *B. siamensis* SB1001 and IR10 with different carbon source based media. Furthermore, batch, fed-batch, and repeated fed-batch fermentations were performed in a 3-L fermentor for γ-PGA production from molasses. The results show the highest γ-PGA productivity compared with previous studies. This strain will serve as a candidate for economical production of γ-PGA on an industrial-scale.

## Methods

### Strains and medium

*Bacillus siamensis* SB1001 was isolated from organically cultivated soybeans and maintained in sterile glycerol (50%, w/v) at - 80 °C [23]. The mutant strain *B. siamensis* IR10 was isolated in this study. Basal medium was used for the growth and pre-culture of the strains. It contained 10 g/L peptone, 5 g/L yeast extract, 10 g/L NaCl, 20 g/L glucose and 20 g/L l-glutamic acid.

The fermentation medium consisted of 30 g/L l-glutamic acid, 5 g/L NH_4_Cl, 2.4 g/L K_2_HPO_4_, 0.5 g/L MgSO_4_·7H_2_O, 0.04 g/L FeCl_3_·6H_2_O, 0.15 g/L CaCl_2_·2H_2_O, 0.104 g/L MnSO_4_·H_2_O, 0.5 g/L NaCl and 30 g/L carbon source (glucose, sucrose, glycerol, et al.). The effect of the molasses on the production of γ-PGA was studied by substituting carbon sources with molasses at 80–140 g/L. The initial pH was adjusted to pH 6.5 ± 0.1 with 1 M NaOH.

The molasses was purchased from Byeoli Science Co., Ltd (Jeonju, South Korea), and it contained 28.96% (w/w) sucrose, 4.58% (w/w) glucose, 7.43% (w/w) fructose, 14.83% (w/w) other carbohydrates, 0.75% (w/w) crude protein, 4.40% (w/w) salt, 7.35% (w/w) ash, 24.16% (w/w) water, 8.13% (w/w) metal ions such as calcium, potassium, sodium, iron, magnesium, and copper, et al. Before using the molasses, it was diluted two times and centrifuged at 9500 rpm for 20 min to remove the undissolved substance.

### Culture condition and fermentation

A loopful of cells from the basal medium agar plate was transferred to 50 mL tubes containing 15 mL basal medium and incubated for 24 h at 37 °C with agitation at 200 rpm. The seed culture was adjusted to OD_600_ to 5.0 ± 0.1, and 1% (v/v) were transferred to 250 mL flasks containing 50 mL of fresh fermentation medium. The flasks were incubated for 24–48 h with shaking at 200 rpm at 37 °C. Each experiment was repeated three times, and data were presented as the mean value.

For bioreactor fermentation, the seed culture (OD_600_ = 5.0 ± 0.1, 1%) was transferred to 500 mL flasks containing 100 mL basal medium. After incubation for 12 h with shaking at 200 rpm at 37 °C, the culture was transferred to a 3-L fermentor (FMT-ST-D03; Bio System Engineering & Machine Company; Korea) with 0.9 L fermentation medium and cultured at 37 °C. The fermentor (diameter 14 cm) was equipped with two Rushton turbines impellers (diameter 7 cm). The bottom impeller was positioned at a height of 3 cm from the fermentor bottom and the top impeller was located at a distance of 7 cm from the bottom impeller. γ-PGA was produced in the batch fermentation with an initial molasses concentration of 100 g/L and 30 g/L l-glutamic acid. Fed-batch fermentation was conducted with an initial molasses concentration of 100 g/L and 60 g/L l-glutamic acid. One hundred grams of molasses treated as above, about 150 mL was added to the bioreactor after sterilization at 121 °C for 15 min when the concentration of the total sugar was lower than 5 g/L. Six repeated fed-batch fermentations were done to investigate the stability of the γ-PGA production. After a 24 h fermentation, the broth was removed retaining only 100 mL and then filled with 900 mL of fresh fermentation medium containing 100 g molasses and 60 g l-glutamic acid. The pH was automatically controlled at 6.5 ± 0.1 by adding NH_4_OH or 1 M HCl in the fermentors. In the batch culture, the aeration and the agitation speed were maintained at 2 L/min and 400 rpm. For the fed-batch, after 12 h, the aeration was increased to 3 L/min, and the agitation speed was adjusted to 600 rpm to maintain the dissolved oxygen above 5%.

### Irradiation and isolation of mutant strains

*B. siamensis* SB1001 was grown in a 250 mL flask containing 50 mL basal medium at 37 °C with shaking at 200 rpm for 12 h. The bacteria solution was transferred to 15 mL centrifuge tubes and exposed to dosages of 1.0, 2.0, and 3.0 kGy in a ^60^cobalt facility (Korea Atomic Energy Research Institute, Jeongup, Republic of Korea) to study the effect of different dosages of ^60^Co γ-rays on microbial lethality. Then, the cells were spread onto basal medium agar plates after suitable dilutions and incubated at 37 °C. The survival curve of *B. siamensis* SB1001 was plotted for the different dosages of gamma irradiation with the colony count method. The radiation dose required to kill above 99.99% of the microorganisms was chosen as the optimal radiation dose. After irradiation at the optimal dose, the colonies with more wrinkles and higher viscosity were selected in contrast with the wild type colonies.

### Analysis of the enzyme activities

*Bacillus siamensis* SB1001 and the mutant strain IR10 were grown in fermentation medium with different carbon sources (sucrose, glucose, fructose and molasses) in flasks for 24 h as described above. The cell extracts were prepared for isocitrate dehydrogenase (ICDH), glutamate dehydrogenase (GDH), α-oxoglutarate dehydrogenase complex (ODHC), glutamate α-oxoglutarate aminotransferase (GOGAT), and glutamate racemase (GLR) assays. The enzyme activities of ICDH, GDH, ODHC, and GOGAT were determined by measuring the appearance or disappearance of NADH or NADPH at 340 nm (ε_340_ = 6.22 mM^−1^cm^−1^) [16, 24]. One unit of enzyme activity was defined as the amount of enzyme catalyzing the formation of 1 μmol NADH or NADPH per min. The GLR enzyme activity was analyzed by the HPLC method [17]. The protein concentration was determined by the Bradford method [25].

### Analysis method

The cell biomass was determined by measuring the absorbance of the broth at 600 nm using a UV–Vis spectrophotometer (Libra S70PC, Biochrom Ltd., Cambridge, England) [23].

The γ-PGA concentration was determined by gel permeation chromatography (GPC) system using an Agilent 1100 high-performance liquid chromatography (HPLC) system equipped with a PL aquagel-OH MIXED-H column (300 × 7.5 mm, 8 μm; Agilent Technologies, Inc., UK) and refractive index detector (RID). HPLC grade water was used as the mobile phase with the flow rate of 1 mL/min and the injection volume of 50 µL. The amount of γ-PGA was calculated from the peak area of the GPC measurements with purified γ-PGA (Bioleaders Corporation, Daejeon, South Korea) as a standard.

Sucrose, glucose and fructose were measured by the HPLC system equipped with an Aminex HPX-87P column (300 × 7.8 mm; Bio-Rad, Hercules, CA, USA). While, l-glutamic acid was measured using a Chirex^®^ 3126 (d)-phenicillamine column (250 × 4.6 mm; Phenomenex Inc., Torrance, CA, USA) and a UV detector (254 nm) as described before [23].

## Results and discussion

### Screening for a high γ-PGA producer in a glucose-based medium

The appropriate dosage rate of ^60^Co γ-ray is important to achieve microbial mutation and to determine the microbial lethality. No colonies were found on the agar plates when the dosage was 3.0 kGy, suggesting that a high dosage of γ-ray had a lethal effect on *B. siamensis* SB1001. In addition, large numbers of colonies formed on the agar plates with a dosage of 1.0 kGy. To screen for a glucose-utilizing, high γ-PGA producing strain, seven mutant colonies whose morphology was mucoid and had more wrinkles than the other colonies were picked from the agar plates. These colonies were exposed to a dosage of 2.0 kGy at the ^60^cobalt facility, and the lethality rates were up to 99.994%. A subsequent comparison of the γ-PGA production by these mutant strains and wild type was done as described above. Among these strains, all the glucose was exhausted within 24 h. IR10 showed the highest γ-PGA production (7.74 ± 0.35 g/L), about 180% higher than that of its parent strain, the growth rate and l-glutamic acid consumption rate were also faster in the glucose-based medium. Besides, IR10 showed the highest conversion rate of l-glutamic acid to γ-PGA (Table 1).

**Table 1:** γ-PGA production by *B. siamensis* SB1001 mutant strains

Strains	12 h		18 h		24 h		Residual l-glutamic acid (g/L)	Conversion rate g_(γ-PGA_)/g(_l-glutamic acid_)
	Biomass (g/L)	γ-PGA (g/L)	Biomass (g/L)	γ-PGA (g/L)	Biomass (g/L)	γ-PGA (g/L)		
SB1001	2.31 ± 0.06	1.37 ± 0.03	3.76 ± 0.11	2.1 ± 0.05	4.49 ± 0.20	2.77 ± 0.07	16.17 ± 0.56	0.21
IR5	3.93 ± 0.11	3.77 ± 0.07	4.02 ± 0.18	5.30 ± 0.22	3.78 ± 0.10	6.62 ± 0.25	14.41 ± 0.62	0.42
IR7	4.06 ± 0.18	5.50 ± 0.23	4.18 ± 0.18	6.03 ± 0.24	4.51 ± 0.18	5.99 ± 0.24	15.66 ± 0.55	0.42
IR8	4.30 ± 0.15	0.89 ± 0.01	4.56 ± 0.16	1.04 ± 0.01	5.34 ± 0.22	1.63 ± 0.01	18.88 ± 0.67	0.15
IR9	5.13 ± 0.20	0.79 ± 0.01	5.19 ± 0.20	1.60 ± 0.03	6.08 ± 0.24	2.18 ± 0.05	14.23 ± 0.52	0.14
IR10	3.96 ± 0.12	5.60 ± 0.23	4.42 ± 0.18	6.86 ± 0.31	4.82 ± 0.21	7.74 ± 0.35	12.32 ± 0.48	0.44
IR12	3.89 ± 0.12	1.67 ± 0.03	5.06 ± 0.23	1.81 ± 0.02	6.24 ± 0.25	1.97 ± 0.02	13.25 ± 0.46	0.12
IR14	4.28 ± 0.16	5.51 ± 0.23	4.42 ± 0.15	5.69 ± 0.24	5.08 ± 0.19	5.79 ± 0.22	13.66 ± 0.51	0.35

*Bacillus siamensis* SB1001 is an l-glutamic acid dependent strain. Glucose provides energy but most of the l-glutamic acid was metabolized as a nitrogen source under the impact of glucose [23]. The mutant strain IR10 improved the conversion rate of l-glutamic acid to γ-PGA, suggesting the activity of enzymes that promote γ-PGA synthesis might be improved. When bacteria are exposed to gamma rays, a large amount of free radicals and reactive oxygen species are produced, which results in some changes in the nucleic acid base sequence of the bacterial genome [26]. The dose rate of 3.0 kGy might exceed the threshold causing serious damage to the *B. siamensis* SB1001 DNA, which leads to cell death [26]. In this study, gamma radiation was applied to *B. siamensis* SB1001 to improve its production of γ-PGA in a glucose-based medium. Previous reports have focused on genetic engineering methods to boost γ-PGA production, such as NADPH regeneration in *B. licheniformis* WX-02 [18], cloning of *pgsBCA* genes in *B. amyloliquefaciens* LL3 [27], chromosomal integration of the *Vitreoscilla* hemoglobin gene (*vgb*) in *B. subtilis* [28], and construction of energy-conserving sucrose utilization pathways in *B. amyloliquefaciens* [12]. This study is the first report that a gamma radiation mutant strain could promote γ-PGA production.

### Effect of carbon sources on γ-PGA production by *B. siamensis* SB1001 and IR10

γ-PGA production by *B. siamensis* SB1001 and the mutant strain IR10 with different carbon sources was investigated in flasks (Table 2). The results show that, IR10 was better in utilizing the different carbon sources for cell growth and γ-PGA synthesis compared with SB1001. It could use all the tested carbon sources except for galactose to produce γ-PGA. A γ-PGA concentration of 7.74 ± 0.35 g/L and a biomass of 4.82 ± 0.21 g/L were obtained in the glucose-based medium, followed by fructose and sucrose as carbon sources with a γ-PGA concentration of 7.21 ± 0.26 g/L and 7.14 ± 0.25 g/L, respectively. Among the three carbon sources, sucrose was more favorable for cell growth, and the highest biomass of 6.22 ± 0.28 g/L was obtained by using sucrose. Molasses consist of glucose, sucrose and fructose, which might be used as an economic carbon source for the economical production of γ-PGA by IR10.

**Table 2:** Effect of carbon sources on γ-PGA production by *B. siamensis* SB1001 and mutant strain IR10

Carbon source	12 h				24 h				Biomass (*P* value)	
	Biomass (g/L)		γ-PGA (g/L)		Biomass (g/L)		γ-PGA (g/L)			
	SB1001	IR10	SB1001	IR10	SB1001	IR10	SB1001	IR10	SB1001	IR10
Glucose	2.31 ± 0.08	3.96 ± 0.12	1.37 ± 0.05	5.60 ± 0.23	4.49 ± 0.18	4.82 ± 0.21	2.77 ± 0.11	7.74 ± 0.35	1.76 × 10^−6^	2.44 × 10^−6^
Fructose	2.69 ± 0.07	4.25 ± 0.13	0.34 ± 0.01	4.99 ± 0.18	4.56 ± 0.17	4.86 ± 0.21	0.36 ± 0.02	7.21 ± 0.26	1.32 × 10^−6^	2.36 × 10^−6^
Sucrose	2.52 ± 0.08	4.71 ± 0.13	5.24 ± 0.23	5.70 ± 0.21	4.07 ± 0.14	6.22 ± 0.28	7.06 ± 0.26	7.14 ± 0.25	9.59 × 10^−7^	2.77 × 10^−6^
Glycerol	1.52 ± 0.05	2.99 ± 0.09	1.95 ± 0.06	3.23 ± 0.12	3.33 ± 0.14	6.67 ± 0.26	3.82 ± 0.15	6.81 ± 0.22	2.15 × 10^−6^	1.56 × 10^−6^
Maltose	2.58 ± 0.08	4.08 ± 0.11	0.33 ± 0.01	3.32 ± 0.11	4.36 ± 0.16	5.97 ± 0.23	0.55 ± 0.03	6.98 ± 0.22	1.24 × 10^−6^	1.49 × 10^−6^
Mannose	0.02 ± 0.01	4.18 ± 0.12	0	4.09 ± 0.15	0.02 ± 0.01	4.80 ± 0.21	0	7.11 ± 0.24	1	2.48 × 10^−6^
Xylose	1.35 ± 0.05	2.47 ± 0.06	0.31 ± 0.01	1.73 ± 0.05	4.36 ± 0.18	5.88 ± 0.22	0.64 ± 0.02	6.22 ± 0.18	1.98 × 10^−6^	1.33 × 10^−6^
Lactose	0.42 ± 0.02	4.46 ± 0.14	0	3.79 ± 0.14	1.38 ± 0.03	5.79 ± 0.20	0	7.09 ± 0.25	1.95 × 10^−7^	9.65 × 10^−7^
Galactose	0.18 ± 0.01	1.27 ± 0.13	0	0	0.51 ± 0.02	3.09 ± 0.15	0	0.72 ± 0.03	2.88 × 10^−6^	3.81 × 10^−6^

Initial biomass (0 h) 0.02 g/L; Statistical analysis was performed for the biomass at 0 h and 24 h. *P*-values < 0.05 showed the significant differences

Gamma-ray irradiation caused random mutations of *B. siamensis*, and the increased production of γ-PGA might indicate mutations in the gene sequences that are expressed in metabolic pathways for different carbon sources. In *B. cereus* B4081 and B4087, presence of a lactose utilization gene cassette, which encodes 6-phospho-β-galactosidase, phosphorylated lactose was hydrolyzed to galactose-6-phosphate and glucose [29, 30]. *B. subtilis* contains the genes which encode the proteins necessary for the degradation of lactose and galactose, and these genes are expressed under inducing β-galactosidase. But the toxic effect of galactose might be occurred due to the accumulation of UDP-galactose [31]. *B. siamensis* SB1001 can slightly grow on lactose and galactose (Table 2), which might be affected by the toxic of UDP-galactose. However, the mutant strain only utilized galactose for cell growth (Table 2), suggesting that galactose metabolism was through the Leloir pathway in *B. siamensis* IR10 [32]. Mannose is first phosphorylated to mannose-6-phosphate by hexokinase and then converted to fructose-6-phosphate by mannose-6-phosphate isomerase [33]. *B. siamensis* SB1001 cannot use mannose (Table 2) because the relative enzymes might not be expressed. Thus, mutations may occur in the genes encoding the enzymes for the lactose, mannose and galactose metabolism pathways.

It was reported that glucose can affect the γ-PGA synthetic enzyme system and catabolite control protein A (CcpA) [34]. CcpA possesses several functions, such as control of lactose transport, β-galactosidase activity, and glycolysis [35], CcpA represses the TCA cycle and activates the EMP pathway when glucose is present in the medium [36]. Additionally, previous research found that a low concentration of glucose was better for the production of γ-PGA by *B. siamensis* SB1001 [23]. Unfortunately, the effect of other carbon sources on γ-PGA synthesis has not been studied. Fructose, maltose, and xylose could support the growth of *B. siamensis* SB1001, but they did not promote γ-PGA synthesis (Table 2). Thus, another mutation in the gene sequence might control the γ-PGA synthetic pathway and its relative proteins. In the future, the whole transcriptomes of the wild and mutant strains need to be compared using mRNA sequencing.

### Effect of the molasses concentration on γ-PGA production by mutant strain IR10

**Fig. 1:**
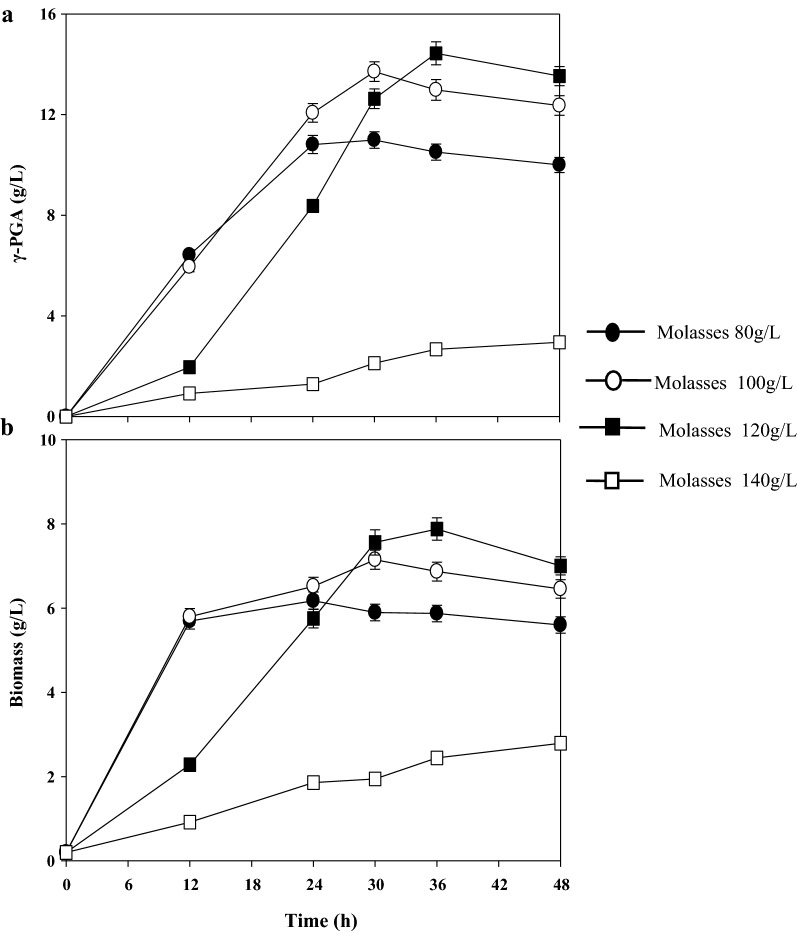
Effect of the molasses concentration for γ-PGA production by *B. siamensis* IR10

Based on the metabolism of the different carbon sources, different concentrations of molasses were chosen for the γ-PGA production. The results in Fig. 1 imply that the molasses was a suitable carbon source for γ-PGA synthesis. γ-PGA concentration and the biomass were increased by increasing the concentration of the molasses from 80–120 g/L. The maximum γ-PGA production of 14.44 ± 0.55 g/L with the productivity of 0.41 ± 0.02 g/L/h and biomass of 8.02 ± 0.38 g/L were obtained at 120 g/L molasses at 36 h. However, the 120 g/L molasses concentration initially showed inhibition during the first 12 h; moreover, a molasses concentration of 140 g/L showed significant inhibition of cell growth and γ-PGA synthesis. A biomass of 7.15 ± 0.36 g/L was obtained at 100 g/L molasses, and the γ-PGA concentration was 13.71 ± 0.51 g/L with the productivity of 0.46 ± 0.02 g/L/h after 30 h; the lower concentration may be caused by the lower sugar concentration in the molasses.

Untreated molasses normally contain some unknown essential nutrients, which could stimulate cell growth [20]. It also contains some metal ions and ash, which could be toxic for cells [37]. When *B. siamensis* IR10 was cultured in medium with over 120 g/L molasses, the number of metal ions and ash exceeded the tolerance limit of strain; thus, γ-PGA synthesis and cell growth were inhibited. A similar phenomenon has been reported for *S. albulus* PD-1, in which the optimum concentration of the untreated cane molasses was 40 g/L. The biomass, poly (ε-l-lysine), and poly (l-diaminopropionic acid) concentrations all decreased when the initial molasses concentration was over 40 g/L [37]. In this study, 100 g/L of initial molasses in the medium was selected for the economic production of γ-PGA by IR10.

### Batch fermentation of untreated molasses for γ-PGA production

**Fig. 2:**
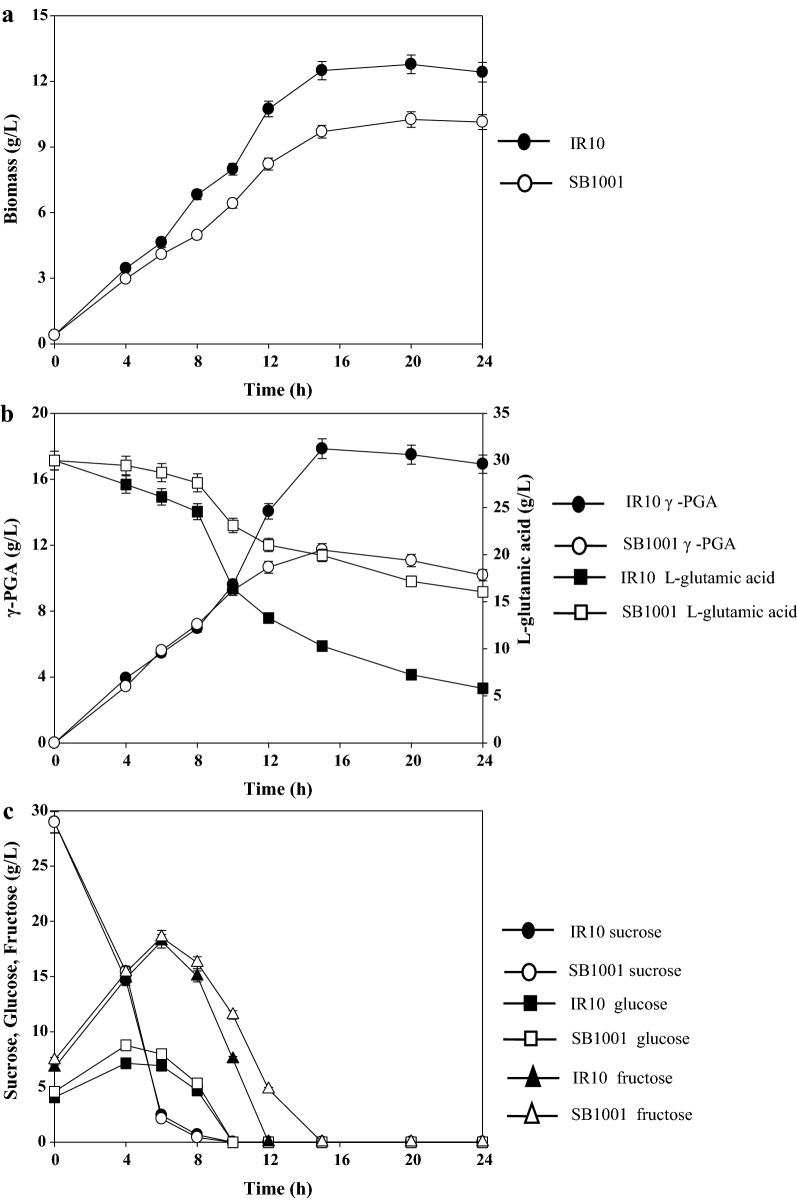
Comparison between *B. siamensis* SB1001 and mutant strain IR10 for γ-PGA production using molasses in the fermentor

To confirm the feasibility of untreated molasses as a sole carbon source for γ-PGA production by the mutant strain, batch fermentation was performed in 3 L fermentors, and the wild type strain was used as the control. Compared with the wild type strain, the mutant strain showed a higher biomass (12.78 ± 0.43 vs 10.26 ± 0.35 g/L) (Fig. 2a), higher γ-PGA yield (17.86 ± 0.97 vs 11.71 ± 0.58 g/L), higher γ-PGA productivity (1.19 ± 0.06 vs 0.78 ± 0.04 g/L/h) and lower residual l-glutamic acid (5.80 ± 0.29 vs 16.05 ± 0.78 g/L) after 24 h (Fig. 2b), suggesting that the mutant strain IR10 had more advantages in producing γ-PGA. Unfortunately, the mutant strain showed a lower conversion rate of l-glutamic acid to γ-PGA (0.91 g _γ-PGA_/g _l-glutamic acid_) than its parent strain (1.16 g _γ-PGA_/g _l-glutamic acid_).

The time course of utilization of sucrose, glucose, and fructose in the untreated molasses is shown in Fig. 2c. Sucrose was rapidly hydrolyzed into glucose and fructose before 8 h, and there was no significant difference in the consumption rates by the two strains. The levels of glucose and fructose first increased in the medium, indicating that *B. siamensis* possesses sucrose-utilization systems. There were obvious differences in the utilization of glucose and fructose between the IR10 and SB1001. The mutant strain could metabolize glucose and fructose much faster. Furthermore, the glucose consumption rate was higher than that of fructose. After the glucose was exhausted at 10 h, the fructose consumption increased, which might be caused by the effects of catabolic repression [38]. Interestingly, all the carbon sources exhausted after 15 h; the two strains continued to consume l-glutamic acid, but the production of γ-PGA did not increase. The phenomenon was consistent with the previous description that l-glutamic acid was metabolized as a nitrogen source to maintain the growth of cells [39]. Sucrose was the best carbon source for *B. siamensis* SB1001 and l-glutamic acid could be converted from sucrose through the tricarboxylic acid cycle [23]. But in *B. siamensis* IR10, the conversion rate of l-glutamic acid to γ-PGA was lower than 1.0 g _γ-PGA_/g _l-glutamic acid_, might be caused by the high biomass consumed more sugars and more l-glutamic acid consumed as a nitrogen source.

### Fed-batch fermentation for γ-PGA production by *B. siamensis* IR10

**Fig. 3:**
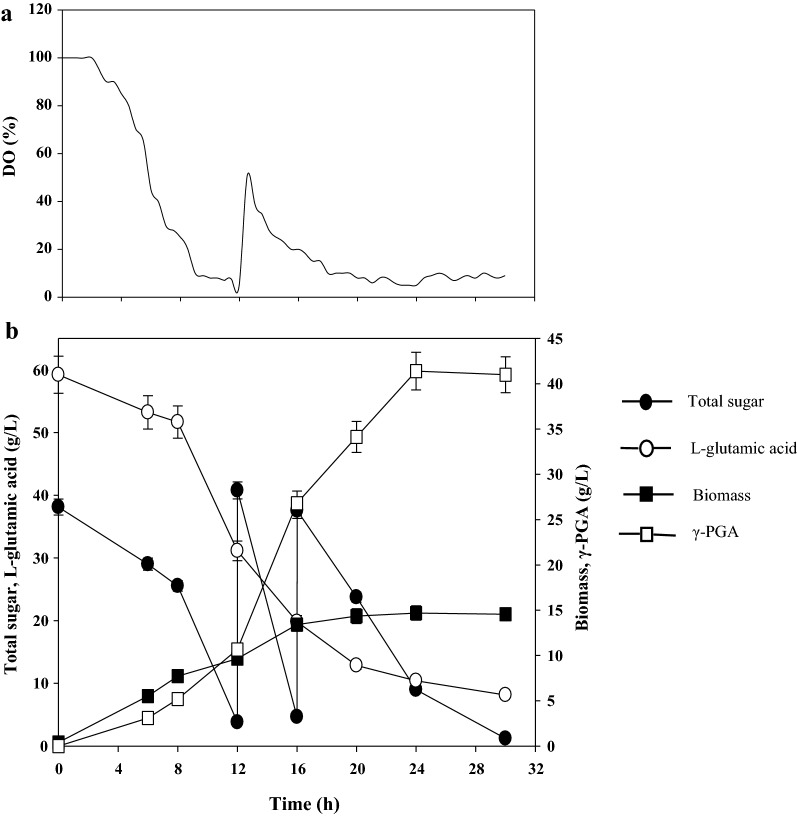
Fed-batch fermentation of γ-PGA using untreated molasses in the fermentor by *B. siamensis* IR10

The batch fermentation results showed that untreated molasses is a potential low-cost carbon source for γ-PGA production by *B. siamensis* IR10. To test the feasibility of an industrial application, the fed-batch fermentation of untreated molasses was performed in a fermentor and the dissolved oxygen level was remained above 5% (Fig. 3a). As shown in Fig. 3b, when the initial concentration of l-glutamic acid was 60 g/L, 41.40 ± 2.01 g/L of γ-PGA were obtained with a γ-PGA productivity of 1.73 ± 0.08 g/L/h, and the highest biomass was 14.69 ± 0.71 g/L during 24 h of cultivation. Furthermore, the conversion rate of l-glutamic acid to γ-PGA up to 101.38% (g _γ-PGA_/g _l-glutamic acid_), suggesting the possible transformation of abundant carbon sources to endogenous glutamic acid.

Some attempts have been made to improve the γ-PGA production with molasses including hydrolysis of the molasses and immobilized cell fermentation [20, 40]. Until now, the highest γ-PGA concentration was 52.1 g/L with a productivity of 0.54 g/L/h with molasses as the carbon source [20]. The γ-PGA productivity in this study is the highest compared to previous reports. The concentration of γ-PGA did not increase after 24 h, which perhaps was caused by the decreased activity of the enzyme controlling the synthesis of γ-PGA. Oxygen plays an important role in γ-PGA synthesis; the production of γ-PGA increases the medium viscosity and thus reduces the mass transport. During fed-batch fermentation, the dissolved oxygen was maintained above 5% (Fig. 3a), meanwhile, we found that further increase agitation speed and aeration did not increase dissolved oxygen and did not increase the γ-PGA concentration (data not show). Previous research reported that dissolved oxygen level remained 5% is the minimum acceptable value and remained high dissolved oxygen (≥ 20%) was beneficial to improve γ-PGA production [41]. But in a bioreactor, increasing the stirring rate and aeration might not be enough to enhance dissolved oxygen, one possible approach was added the oxygen carriers [42]. Another reason might be that a high concentration of molasses contains more harmful substances that exceed the tolerance ability of the strain. Further optimization of the fermentation process to enhance the γ-PGA yield should be carried out. Anyway, molasses can be efficiently utilized in γ-PGA production by *B. siamensis* IR10, which may contribute to the commercial-scale production of γ-PGA by industry byproducts.

### Repeated fed-batch fermentation for γ-PGA production by *B. siamensis* IR10

**Fig. 4:**
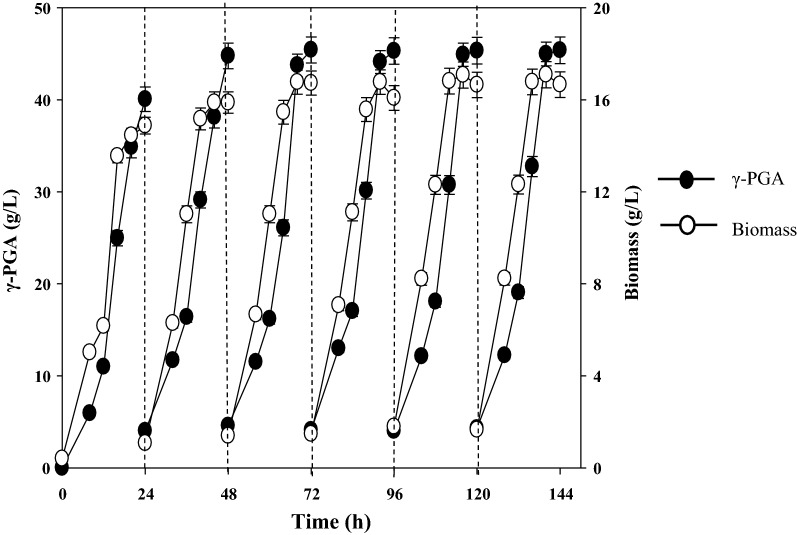
Repeated fed-batch fermentation of γ-PGA using untreated molasses in the fermentor by *B. siamensis* IR10

To investigate the long-term stability and maximize the γ-PGA production, repeated fed-batch fermentations were done in this study. This approach has been widely used to improve the productivity of γ-PGA because it reduces the delay period of the strains [40, 43]. Based on the fed-batch results, we performed repeated fed-batch fermentations of *B. siamensis* IR10 for six cycles each 24 h long (Fig. 4). As shown in Table 3, the cell growth and γ-PGA productivity increased as the batch number increased.

**Table 3:** Comparison of parameters of each cycle for γ-PGA production by *B. siamensis* IR10 in repeated fed-batch fermentation

Cycle number	1	2	3	4	5	6
Time (h)	24	24	24	20	20	20
Biomass (g/L)	14.88 ± 0.49	15.88 ± 0.52	16.73 ± 0.55	16.77 ± 0.56	17.08 ± 0.57	17.09 ± 0.56
γ-PGA concentration (g/L)	40.06 ± 1.33	44.77 ± 1.39	45.42 ± 1.41	44.10 ± 1.23	44.90 ± 1.26	44.98 ± 1.26
γ-PGA productivity (g/L/h)	1.67 ± 0.05	1.70 ± 0.06	1.71 ± 0.06	1.98 ± 0.06	2.02 ± 0.06	2.02 ± 0.06

The productivity was calculated according to the produced γ-PGA production as follows

Cycle 1: 40.06 ÷ 24 = 1.67; Cycle 2: (44.77 − 4.00) ÷ 24 = 1.70; Cycle 3: (45.42 − 4.40) ÷ 24 = 1.71; Cycle 4: (44.10 − 4.54) ÷ 20 = 1.98

Cycle 5: (44.90 − 4.53) ÷ 20 = 2.02; Cycle 6: (44.98 − 4.53) ÷ 20 = 2.02

In the first cycle, the biomass reached 14.88 ± 0.49 g/L and then increased each cycle and finally stayed constant at 17.08 ± 0.57 g/L in the last two cycles. From first to the last cycle, the γ-PGA concentration increased from 40.06 ± 1.33 g/L to 45.42 ± 1.36 g/L and stayed constant at about 45 g/L. During the repeated fed-batch fermentation, the high γ-PGA productivity (1.67–2.02 g/L/h) was successfully maintained with an average of 1.85 g/L/h. The results suggest that γ-PGA production in the repeated fed-batch was highly stable and showed prominent advantages. Several strategies have been made to improve γ-PGA production and productivity shown in Table 4. *B. subtilis* NX-2 had the highest yield of γ-PGA (74.2 g/L) with a productivity of 1.24 g/L/h [43]. However, their γ-PGA productivity was much lower than the value of 1.85 g/L/h observed in the present study. Although the repeated fed-batch in this study does not provide the highest γ-PGA concentration, the γ-PGA production rate is the highest and more economical with molasses as the carbon source.

**Table 4:** Comparison of γ-PGA production and productivity in the fermentor

Strain	Key nutrients	Fermentation process	Production (g/L)	Productivity (g/L/h)	References
*B. licheniformis* NCIM 2324	l-Glutamic acid, citric acid, sugarcane juice, NH_4_Cl	Membrane-integrated hybrid reactor system (30 L), continuous feed fermentation	36.5	0.91	[49]
*B. licheniformis* P-104	Sodium glutamate, glucose, sodium citrate,(NH_4_)_2_SO_4_	Fed-batch fermentation (7 L)	41.6	1.07	[50]
*B. subtilis* NX-2	Glutamate, glucose, (NH_4_)_2_SO_4_	Aerobic plant fibrous-bed bioreactor (7.5 L), repeated fed-batch fermentation	71.21	1.246	[40]
*B. subtilis* BL53	Glutamic acid, citric acid, glycerol, NH_4_Cl	Bioreactor (5 L) with oxygen carrier, batch fermentation	23.5	0.98	[42]
*B. subtilis* NX-2	Glutamate, glucose, (NH_4_)_2_SO_4_	Moving bed biofilm reactor (7.5 L), repeated fed-batch fermentation	74.2	1.24	[43]
*B. siamensis* IR10	l-Glutamic acid, molasses, NH_4_Cl	Fed-batch and repeated fed-batch fermentation (3 L)	41.4–45.42	1.67–2.02	This work

### Enzyme activity analysis around the 2-oxoglutarate branch in *B. siamensis* SB1001 and IR10

*B. siamensis* IR10 showed good advantages in utilizing glucose, fructose, and molasses for the production of γ-PGA. Although different carbon sources have different metabolic pathways, the pathway to γ-PGA synthesis is unique, which is around the 2-oxoglutarate branch. Thus, we analyzed the effect of different carbon sources on the activities of enzymes around the 2-oxoglutarate branch.

**Fig. 5:**
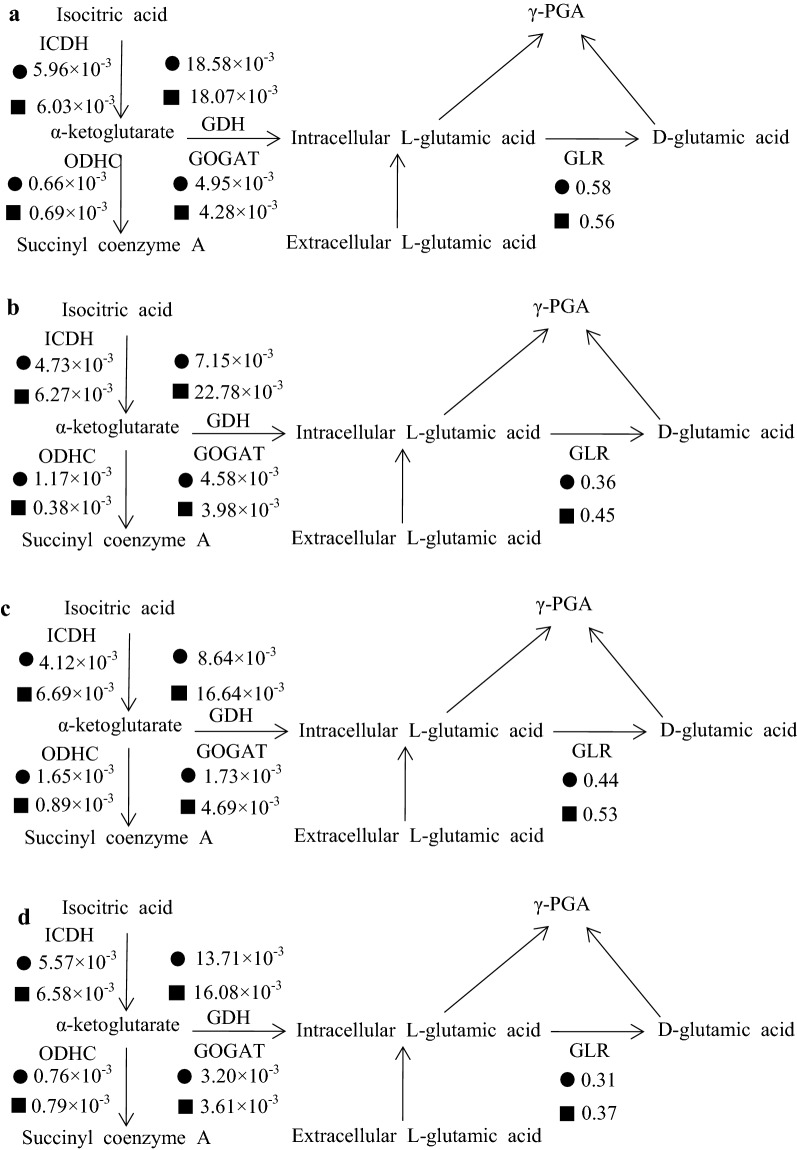
Enzyme activities in ● *B. siamensis* SB1001 and ■ *B. siamensis* IR10 with **a** sucrose, **b** glucose, **c** fructose and **d** molasses as the carbon source

As shown in Fig. 5, the enzyme activities of ICDH, ODHC, GDH, GOGAT, and GLR were different in *B. siamensis* SB1001 and *B. siamensis* IR10 when cultured in different carbon sources. There was no significant difference in the tested enzyme activities in a sucrose-based medium (Fig. 5a); thus, it did not affect the production of γ-PGA between the wild type and mutant strain (Table 2). The activities of ICDH increased from 4.73 × 10^−3^ to 6.27 × 10^−3^, 4.12 × 10^−3^ to 6.69 × 10^−3^, and 5.57 × 10^−3^ to 6.58 × 10^−3^ U/mg when cultured in a glucose-, fructose-, and molasses-based medium, respectively. Meanwhile, the activities of GDH increased from 7.15 × 10^−3^ to 22.78 × 10^−3^, 8.64 × 10^−3^ to 16.64 × 10^−3^, and 13.71 × 10^−3^ to 16.08 × 10^−3^ U/mg (Fig. 5b–d), respectively. In addition, the activity of ODHC in *B. siamensis* IR10 decreased by more than 3- and 1.8-fold compared to the wild type strain when glucose and fructose were the carbon sources, respectively. The activities of GOGAT and GLR increased in *B. siamensis* IR10 when only cultured in the fructose-based medium.

For *B. siamensis* IR10, ICDH and GDH had a higher activity compared with the wild type strain when cultured in the glucose- and fructose-based medium. This result shows that ICDH and GDH had positive regulation for γ-PGA production in *B. siamensis* SB1001, which is consistent with *B. subtilis* GXA-28 [16]. According to a previous report, GOGAT showed a greater contribution than GDH to the conversion of α-oxoglutarate to glutamate [44]. In the current study, the contributions of the two enzymes were not compared. Both the wild type and mutant strains had a low activity of ODHC when producing a high yield of γ-PGA, suggesting ODHC has the most important role in controlling the flux distribution at the 2-oxoglutarate branch in *B. siamensis* SB1001. Previous research found that ODHC was the greatest impact factor on glutamate production, and glutamate production was markedly increased by the attenuation of the ODHC activity [45]. Interestingly, the carbon sources can affect the enzyme activities, the regulation could take place at the level of the CcpA response to the different carbon sources and mixtures. CcpA is a key factor in the regulation of carbon metabolism, carbon sources can affect the complex of CcpA represses many genes and operons for regulation TCA cycle and control of nitrogen source metabolism [46]. Also, CcpA plays an important role in the regulation of metabolism for γ-PGA synthesis, GOGAT and GDH activities were regulated by CcpA in the presence of different carbon sources in *Bacillus* sp. [47]. Another reason perhaps caused by the redox reaction involved with NADP^+^ and NADPH, which shows an imbalance in the central carbon metabolism, which could affect the TCA cycle and amino acids metabolism [48].

## Conclusion

In this study, a mutant strain *B. siamensis* IR10 was screened successfully using the ^60^Co γ-ray mutation method. A high γ-PGA concentration and productivity were obtained with a molasses-based medium in fed-batch fermentation. Furthermore, a high average γ-PGA productivity of 1.85 g/L/h was achieved, which exhibited long-term stability in repeated fed-batch fermentation. *B. siamensis* IR10 showed great advantages in producing γ-PGA from different carbon sources, caused by the increased activity of ICDH and GDH and the decreased activity of ODHC. This study showed that *B. siamensis* IR10 is a potential γ-PGA producer at an industrial scale using a low-cost medium.

## Data Availability

All data generated or analyzed during this study are included in this article.

## Abbreviations

γ-PGA: Poly-γ-glutamic acid
*B. siamensis*: *Bacillus siamensis*
ICDH: Isocitrate dehydrogenase
GDH: Glutamate dehydrogenase
ODHC: α-Oxoglutarate dehydrogenase complex
GOGAT: Glutamate α-oxoglutarate aminotransferase
GLR: Glutamate racemase
HPLC: High-performance liquid chromatography
GPC: Gel permeation chromatogram
RID: Refractive index detector
